# Richmond Academy of Medicine and Surgery

**Published:** 1900-04

**Authors:** 


### SOCIETY REPORTS.

### RICHMOND ACADEMY OF MEDICINE AND SURGERY.

Regular meeting held February 27, 1900, with William T. Oppenhimer, M.D., President, in the chair; Mark W. Peyser, M.D., Reporter.

Dr. Lewis Wheat read a paper on "Latent Gonorrhea in the Male as a Factor in Diseases of the Female Organs of Generation." (See page 21.)

### DISCUSSION.

Dr. J. S. Wellford remarked that when Noeggerath first stated his views of the effect of gonorrhea in the husband on the wife, he thought them very extravagant; but subsequent information had to a greet degree established their accuracy. Indeed, in this respect and others, he thought that in a healthy subject he would prefer to treat a case of syphilis rather than one of gonorrhea, as in a person easily managed the complications and sequelæ were frequently not so serious. When gonorrhea in the female was confined to the vagina, the treatment was comparatively easy; but he would request Dr. Wheat to give an outline of what he regarded as best when the uterus, ovaries and tubes were involved.

Dr. Stuart McGuire stated that so far as he could remember he had recognized in his practice but one case of acute gonorrhea in a woman. Doubtless many individuals suffering from the disease had consulted him, and had been advised to use some antiseptic douche for supposed leucorrhea. When these patients came to him it was usually in the last stage of the disease, when its results or sequelæ were the indications for surgical intervention. In the future he would give this class of cases more careful attention, and his treatment would be determined by microscopic examination of the discharges.

Dr. Hugh M. Taylor said that, in his experience, the majority of pelvic troubles was due more to septic parturition than to gonorrhea; and that these troubles were as usual to-day as in the pre-aseptic era. He did not allude to the gross, but to the minor infection, which was more common than we were led to suppose.

Dr. W. W. Dunn said that where urethritis accompanied vaginitis, it was well, besides using permanganate douches, to keep the vaginal walls separated by an aseptic packing that would absorb the discharge.

Dr. Wheat, in concluding the discussion, said that as gonococci frequently remained latent for years in the posterior urethra, it was exceedingly difficult to tell at what time marriage was safe. To determine this, he advised injection of nitrate of silver solution, which would stir up the old inflammation if present. The number of women consulting him for gonorrhea in proportion to men was small; in fact, vaginitis or urethritis in women was very uncommon even in prostitutes. If recognized, it was only after it had attacked the tubes, or more rarely, the uterine mucosa. The germs might remain latent in the vagina even through successive pregnancies, and then take on new life. This solved Dr. Taylor's problem as to the minor conditions in the parturient state. Lydston stated that the normal germs of the vagina, by a species of evolution, might produce gonorrhea in the male urethra. Dr. Wheat said that rarely or never had he seen a virulent case of gonorrhea in the male that was uninfected by streptococci; and that the joints of gonorrheal patients took on septic trouble more readily than when infected with any other malady, except tuberculosis. He thought the best treatment was that which advocated thorough scouring of the cervix, followed by its dilatation; scouring the uterus, curetting it, washing out with bichloride of mercury solution 1-2000, and packing with iodoform or moist bichloride gauze, distending it as much as possible. After these procedures the vagina was thoroughly scoured and lightly packed.

### REPORTS OF CASES.

Dr. Stuart McGuire reported the following cases:

CASE I. *Nephrotomy for Renal Calculus.*—Mrs. L., aged 32 years, for the past two years had suffered with constant ache in the right lumbar region, and on three or four occasions there had been acute exacerbations closely simulating nephritic colic. Clinical symptoms pointed to a calculus in the kidney; and Dr. H. Stuart MacLean was called in consultation to verify or controvert the diagnosis by chemic and microscopic examination of the urine. The latter, after investigation, stated that, while he could not positively say there was renal calculus, he felt sure of its presence, basing his opinion upon the constant presence of renal débris, a small amount of albumin, and a large quantity of pus in strongly acid urine. Two separate attempts were made to get an X-ray picture of the kidney, both times with unsatisfactory results. Despite the uncertainty of the diagnosis, the patient consented to an operation, which was done by him with the assistance of Dr. Peple. The kidney was readily reached and brought out, and palpation and exploratory puncture with a needle located a stone in one of the calices. This was removed through an incision made in the convex border. Bleeding was slight and easily controlled by deep and superficial sutures. Recovery had been uneventful, the only untoward symptom developed being pain due to the passage of blood-clots from the pelvis of the kidney to the bladder. The stone, in size, shape and appearance, closely resembled in Indian arrowhead, and was composed of a uric acid nucleus around which had been deposited phosphatic salts.

CASE II. *Abdominal Nephrectomy for Hydronephrosis.*—Miss W., aged 23, had a history of attacks of vague pain in the abdomen eight years ago, which was followed by the formation of a lump that had gradually increased in size until it produced considerable deformity. Urinary examination revealed no renal disease, the only abnormality being the existence of an unusually large amount of phosphates. Physical examination showed a movable fluctuating tumor in the lower left part of the abdomen. It was about the size of a child's head. An accurate diagnosis being impossible, an exploratory operation was advised. This was agreed to, and was performed by Dr. Hunter McGuire. Through a median incision made below the umbilicus, the tumor, covered by the posterior layer of the peritoneum, at once presented itself, and was seen to originate from the kidney. It was rapidly shelled out, and laid upon the skin of the abdomen. The ureter was ligated, divided, disinfected and returned to the cavity. The small pedicle which remained was tied in sections and cut off, and the abdominal incision then closed. The tumor, which was exhibited to the Academy, was a beautiful illustration of hydronephrosis due to occlusion of the ureter by an impacted calculus. The stone, as could be plainly felt, was lodged in the lumen one and one-half inch below the point of emergence of the ureter from the pelvis of the kidney.

Dr. Gordon asked Dr. McGuire upon what Dr. MacLean based his diagnosis. Pus and acid urine were sometimes found in cystitis limited to the neck of the bladder and in pyelitis from other sources than calculi. It was well known, also, that the epithelium from the pelvis of the kidney and that from other portions of the urinary tract could not always be differentiated. He cited the case of a man now under his care who, in the past, had suffered from several severe attacks of renal colic, so called, and who claimed to have passed brick-dust deposits. Hematuria subsequently appeared, increased by exertion and relieved by copious draughts of lithia water. Pain in the back was now almost constant. Was the cessation of the hemorrhage due to dilatation of the pelvis, or to what? He had not detected uric acid under the microscope.

Dr. McGuire, in reply to a question from Dr. Gordon as to when a physician, knowing or suspecting the existence of a calculus in the kidney, was justified in regarding the case as surgical and asking for a consultation with a view to an operation, stated that every case must be judged for itself; that frequently a calculus in the kidney, after giving trouble for two or three weeks, would be passed through the ureter, and the patient be cured; that where a calculus remained for a longer time in the kidney, causing considerable pain and evincing through the urine signs of the production of irritation and inflammation of the kidney, he believed an operation was imperatively demanded; that an uncomplicated nephrectomy for stone in the kidney was followed by a mortality of less than two per cent., and he believed it would be much better possibly to subject a patient to an unnecessary operation when the risk was so small, than probably to allow a stone to remain in the kidney when the risk was so great, as it would inevitably produce destruction and disintegration of the organ, and ultimately terminate in death. Dr. McGuire then spoke of the additional evidence gained by urinary examination, X-ray photographs and the introduction of an ureteral bougie tipped with wax.

Dr. W. H. Parker reported the following case of aortic aneurism. The patient was a man aged 61 years, anemic and thin, having lost sixty-one pounds in the last year. When first seen the recti muscles were so tense that no thorough examination could be made. Three or four months before he had passed pus from the urethra. Numbers of diagnoses had been made by himself and other physicians, among them being floating kidney, sarcoma of the kidney, of the intestines, tubercular kidney, etc. The patient was sent to the Sheltering Arms Hospital for operation, and the day before it was done Dr. Parker diagnosed abdominal aneurism. Upon opening the abdomen, with the assistance of Dr. W. W. Dunn, a large tumor was seen. It had no bruit, and he inserted a hypodermic needle, but no fluid was withdrawn. A trocar was then introduced, precaution being taken to prevent hemorrhage, and blood came forth. The aperture was then closed without the loss of additional blood, the abdomen sutured, and the patient put to bed. Nothing eventful occurred till ten days later, when a stitch-hole abscess developed that extended downward to the iliac crest, then upward and backward to the ribs. This was treated by Dr. Dunn, and the patient seemingly progressed well till three or four nights ago, when he died. Death was due to hemorrhage produced by the abscess eroding either the superficial or one of the mesenteric vessels. Upon post-mortem examination it was seen that the aneurism extended from the celiac axis to the bifurcation of the aorta, and that it had eroded the vertebræ for a space one-fourth of an inch deep, two inches long and one inch wide. This was thought to be the cause of the intense pain which the patient had suffered for some time, and which had induced him to seek relief. The aneurism had not burst.

Dr. Taylor said that, while the physical signs of the earlier stage of aneurism were not present in this case, those of the later stage were, the absence in the former instance being due to the presence of a laminated clot. He saw the patient in consultation, after the exploratory had been made, and decided that the case was an operable one. His idea was to obliterate the aorta by occluding it so gradually with a ligature that collateral circulation would have been established by the time occlusion had been completed. He believed that this could be and would be done. Dr. Keen, with a few strands of floss silk, had tied the aorta for forty-eight days. In his next operation he would use silk tape, which was both stronger and broader.

